# Plant Mitophagy in Comparison to Mammals: What Is Still Missing?

**DOI:** 10.3390/ijms22031236

**Published:** 2021-01-27

**Authors:** Kaike Ren, Lanlan Feng, Shuangli Sun, Xiaohong Zhuang

**Affiliations:** Centre for Cell and Developmental Biology, State Key Laboratory of Agrobiotechnology, School of Life Sciences, The Chinese University of Hong Kong, Hong Kong, China; 1155136084@link.cuhk.edu.hk (K.R.); 1155118579@link.cuhk.edu.hk (L.F.); sunshuangli@link.cuhk.edu.hk (S.S.)

**Keywords:** mitochondrial homeostasis, mitophagy, mitochondrial fission, mitochondrial fusion, programmed cell death

## Abstract

Mitochondrial homeostasis refers to the balance of mitochondrial number and quality in a cell. It is maintained by mitochondrial biogenesis, mitochondrial fusion/fission, and the clearance of unwanted/damaged mitochondria. Mitophagy represents a selective form of autophagy by sequestration of the potentially harmful mitochondrial materials into a double-membrane autophagosome, thus preventing the release of death inducers, which can trigger programmed cell death (PCD). Recent advances have also unveiled a close interconnection between mitophagy and mitochondrial dynamics, as well as PCD in both mammalian and plant cells. In this review, we will summarize and discuss recent findings on the interplay between mitophagy and mitochondrial dynamics, with a focus on the molecular evidence for mitophagy crosstalk with mitochondrial dynamics and PCD.

## 1. Introduction

The mitochondrion is an essential organelle conserved in eukaryotic organisms, which conducts several key biological functions. The primary function of mitochondria is to manufacture ATP, the major energy resource of the cells, through oxidative respiration reactions [1]. In addition, mitochondria are versatile participants in many catabolic and signaling pathways. For example, studies in animals have established an essential role of mitochondria in fatty acid synthesis for lipoic acid production, mitochondrial translation and mitochondrial oxidative reaction [2]. Another conserved function of mitochondria is to store Ca^2+^ ions to maintain Ca^2+^ homeostasis [3]. In plants, recent findings have also identified a crucial role of mitochondria in mediating salicylic acid signaling [4], multiple anti-stress [5] and anti-pathogen responses [6]. In addition, mitochondria are indispensable for programmed cell death (PCD) regulation in both animals [7] and plants [8]. As a highly active factory where redox reactions occur, mitochondria also produce a lot of reactive oxygen species (ROS), such as H_2_O_2,_ which can damage themselves as well as other organelles [8]. Accumulating ROS in the mitochondrion will ultimately lead to a loss of mitochondrial membrane potential difference (also known as ΔφΜ) and increase its mitochondrial outer membrane permeability (MOMP) [9]. As a result, mitochondrial materials (e.g., cytochrome C), which will activate downstream PCD machinery, are released from the damaged mitochondria [9]. Thus, it is crucial for the cells to surveil the conditions of the mitochondria and to eliminate those damaged/unwanted ones efficiently by regulating mitochondrial dynamics [10].

The overall balance in the quantity and the quality of mitochondria in a cell is referred to as mitochondrial homeostasis maintenance. Mitochondrial fission/fusion and autophagy-selective degradation (also known as mitophagy) represent two major approaches to balance the number and size of mitochondria, or to eliminate the unwanted ones [11,12]. Through the mitochondrial fission/fusion, mitochondria are organized into a huge dynamic network, which increases their anti-stress ability as the mildly damaged mitochondria can be repaired by fusing with healthy ones, while the severely damaged parts of mitochondria can be segregated from the network by fission [13]. On the other hand, the segregated damaged parts of mitochondria can be selectively degraded via mitophagy [14]. During mitophagy, unwanted mitochondrial materials are recognized and enclosed by a double-membrane structure named an autophagosome, which is finally delivered into the lysosome (in animal) or vacuole (in plant and yeast) for degradation [15].

Five core autophagy-related (ATG) complexes are required to aid the formation of an autophagosome, which are largely conserved in plant and animal genomes: (1) ATG1 (ortholog of ULK1 [unc-51 like autophagy activating kinase 1] in animals) kinase complex; (2) ATG9 complex; (3) ATG6 (ortholog of Beclin1 in animals)-VPS15-VPS34 PI3K (phosphatidylinositol 3-kinase) complex; (4) ATG5-ATG12 conjugate; (5) ATG8 (ortholog of LC3 in animals) conjugate [16]. Among them, only ATG8 covalently binds to phosphatidylethanolamine (PE) on the autophagosome membrane, and therefore widely serves as a reporter of autophagosome structures and autophagy activity. The function of the core ATG complexes in autophagosome formation have been extensively summarized in a number of recent reviews, and thus will not be covered here [16,17,18,19].

In recent decades, exciting new findings have unveiled a critical role of mitophagy in plant development. In germinating Arabidopsis seedlings, it has been observed that inactive mitochondria are engulfed by autophagosomes, suggesting that mitophagy facilitates the degradation of mitochondria that fail to recover from dormancy [20]. Recently, it has been also demonstrated that the de-etiolation process is suppressed in *atg5* mutant plants during the transition from darkness to light [21]. In addition, intact mitochondria were observed in the vacuole of *Arabidopsis* under oxidative stress, implying the occurrence of mitophagy in response to stress [15]. Furthermore, mitophagy has also been implicated to participate in PCD regulation and senescence under nutrient deficiency conditions [12,22]. Importantly, recent studies in animal have unveiled a close interconnection between mitophagy and mitochondrial dynamics, as well as PCD, for a balance in mitochondrial quality control mechanisms. Here we will highlight these recent findings to compare with the plant system, with a focus on the interplay among mitophagy regulators, mitochondrial fusion/fission machineries and PCD regulators.

## 2. Mitophagy: More than One Way to Get Rid of a Mitochondrion

**Figure 1:**
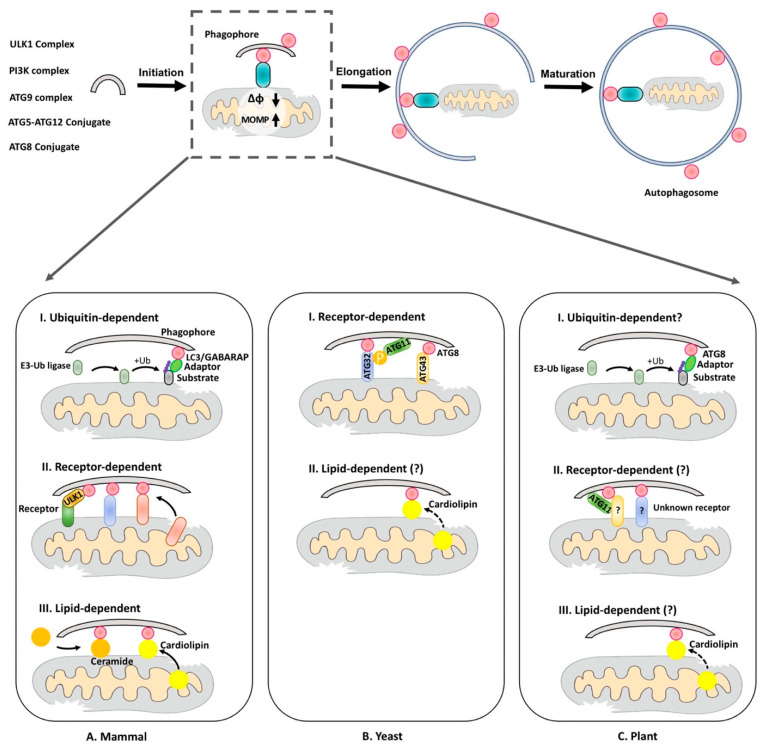
Mitophagy pathways in different organisms. Upon stress damage, the ΔφΜ of the mitochondrion is decreased and the mitochondrial outer-membrane permeability (MOMP) is increased. This will lead to mitophagy, which involves the recruitment of the core autophagy-related (ATG) complexes, initiation of phagophore formation, recognition and sequestration of the unwanted mitochondrial materials into the autophagosome. Three types of mitophagy have been described based on the nature of the mitochondrial proteins recognized by the ATG proteins: (**A**) In animals, mitophagy can be executed through the ubiquitin-dependent, the receptor-dependent and the lipid-dependent pathways. In the ubiquitin-dependent pathway, an E3-Ub ligase is activated and poly-ubiquitinate outer mitochondrial membrane (OMM) substrates, which further recruit adaptor proteins to link the poly-Ub chain and LC3 [24,25,26,27,28,29]; in the receptor-dependent pathway, the receptor proteins directly bind to LC3 to recruit the phagophore [30,31,32,33,34,35,36,37,38], or form a complex together with ULK1 and LC3 [39]; in the lipid-dependent pathway, the lipids interact with LC3, such as cardiolipin [40] and ceramide [41]. (**B**) In budding yeast, the only identified receptor is ATG32, which is phosphorylated and recognized by ATG11 or ATG8 respectively [42,43,44,45]. Another mitochondrial outer membrane protein, ATG43, serves as the mitophagy receptor in fission yeast [46]. However, the function of cardiolipin is not clear in yeast mitophagy. (**C**) In plants, only ATG11, which binds to ATG8 directly, has been reported to participate in mitophagy regulation [22]. E3-Ub ligase family members as well as cardiolipin synthase have also been identified in plants to regulate mitochondria biogenesis, but whether they also participate in plant mitophagy remains unknown (question marks).

During mitophagy, two key events are involved: (1) the formation of the phagophore, which finally expands and encloses the cargo into the autophagosome; (2) the recognition of the mitochondrial cargo, which is mainly mediated by interaction between the mitochondria cargo receptor/adaptor and ATG8/LC3, which are covalently linked to phosphatidylethanolamine (PE) on the autophagosome membrane [23] (Figure 1). According to the nature of the targeting mitochondrial proteins, mitophagy can be classified into the following categories: (1) ubiquitin-dependent, (2) receptor-dependent and (3) lipid-dependent (Figure 1).

### 2.1. Ubiquitin-Dependent Mitophagy

In humans, the first pathway identified to mediate mitophagy was the PINK1–Parkin system, which is involved in Parkinson’s disease [47]. When mitochondria lose their ΔφΜ induced by ROS, PTEN-induced kinase 1 (PINK1) kinase proteins accumulate on the outer mitochondrial membrane (OMM) to recruit and activate Parkin [48]. Parkin, which is featured by its RING1-in-between-ring (IBR)-RING2 domains, is a ring-in-between-ring (RBR) E3 ubiquitin ligase to ubiquitinate multiple OMM proteins [49]. Afterwards, some adaptor proteins, such as p62, recognizes the poly-ubiquitinated mitochondrial OMMs and targets them to the autophagosome via binding to LC3 [24]. In addition, emerging evidence has indicated that other E3-ubiquitin ligases may participate in mitophagy. For example, mitochondrial ubiquitin ligase activator of NFKB 1 (MUL1/MAPL/MULAN) functions as an alternative OMM-localized E3 ligase in PARKIN-independent mitophagy [25]. UKL1, as well as dynamin-related protein 1 (DRP1), have both been identified as the substrates of MUL1/MAPL/MULAN via ubiquitination and SUMOylating, respectively [26,27]. Another E3 ligase, SIAH1, forms a complex with Synphilin1, which interacts with PINK1 and is recruited to the OMM, to ubiquitinate mitochondrial proteins in a PINK1-Parkin-independent manner [28]. Moreover, a recent study identified another alternative PRB E3 ligase called ARIH1/HHARI, to mediate the mitophagy pathway together with PINK1 in a Parkin-independent manner [29].

In plants, counterparts of these E3-ligases have not yet been identified to date. Instead, the Arabidopsis ARI family, a putative RBR E3-ligase family, has been suggested to play an essential role under stress conditions, and actively participates in several stress-related hormone signaling pathways via the ubiquitin proteasome system [50]. Recently, an RBR E3-ligase ubiquitin conjugating enzyme 26 (UBC26) has been reported to participate in the phytohormone abscisic acid (ABA) response in Arabidopsis, likely by promoting the degradation of ABA receptors in response to abiotic stress [51]. However, direct evidence for linking RBR E3-ligases in plant mitophagy is still missing. Recently, it was reported that another plant E3 ligase, SP1, a counterpart of mammalian MUL1/MAPL/MULAN, which participates in chloroplast and peroxisome biogenesis, also targets to mitochondria [52]. It might be worthwhile to identify the mitochondrial substrates for these E3 ligases during mitophagy in future to further unveil their possible roles in mitochondrial homeostasis.

### 2.2. Receptor-Dependent Mitophagy

Despite the ubiquitin-dependent pathway, ATG8/LC3 also directly binds to several OMM receptors which contain the conserved ATG8-interacting motif/LC3-interacting region (AIM/LIR). OMM receptors such as FUN14 domain-containing protein 1 (FUNDC1) [30], BCL2 Interacting Protein 1 (BNIP1) [32] and NIX [31], are phosphorylated and activated upon mitochondrial damage to recruit LC3 or gamma-aminobutyric acid receptor-associated protein, another LC3 isoform (GABARAP) for binding to different mitochondrial OMM proteins respectively. Recently, a novel receptor named FKBP Prolyl Isomerase 8 (FKBP8) has been identified, but it is recycled from the mitochondria once mitophagy is activated [33]. Interestingly, it has been reported that Bcl2-L-13, functions as another mitophagy receptor to recruit both ULK1 and LC3B to regulate mitophagy [39]. Additionally, an OMM receptor activating molecule in BECN1-regulated autophagy protein 1 (AMBRA1), by recruiting Beclin1, induces PINK1/Parkin-dependent mitophagy pathway [34] and the receptor-dependent pathway [35]. On the other hand, some inner mitochondrial membrane (IMM) mitophagy receptors have also been reported. For example, prohibitin 2 (PHB2) was found to be exported to the OMM for directly binding to LC3 to stimulate mitophagy [36]. Differently, another IMM protein, choline dehydrogenase (CHDH), is initially recognized by p62 adaptor and then recruits LC3 [37]. Moreover, it has also been reported that E3 ligase MUL1/MAPL/MULAN contains an LIR-like motif to interact with GABARAP, another LC3 isoform in animals, implying that E3 ligases may alternatively participate in mitochondrial turnover in a receptor-dependent manner as well [38].

In budding yeast, the only identified receptor is ATG32, an OMM-localized protein interacting with both ATG8 and ATG11 [42]. Upon mitophagy induction, casein kinase 2 (CK2) will activate ATG32 via phosphorylation. Subsequently, the phosphorylated ATG32 is recognized by ATG11 [43]. Meanwhile, ATG32 also requires an IMM protease FtsH/Yme1 for its C-terminus processing in order to interact with ATG11 [44]. Although ATG32 contains an AIM motif for binding to ATG8, mutation of ATG32–ATG8 interaction dose not completely block mitophagy [45]. On the other hand, ATG11 binds to Dynamin 1 (DNM1), which functions in mitochondrial fission [53]. However, it remains unknown whether the ATG32–ATG11 complex promotes mitochondrial fission by interacting with DNM1. Differently, in fission yeast, it has been found that another OMM protein, named ATG43, might function as a mitophagy receptor by coordinating with the mitochondrial import factors instead [46].

In plants, ATG11 has also been suggested to function as an adaptor in autophagy-dependent mitochondrial degradation [22]. Arabidopsis ATG11 contains AIM for interacting with ATG8, and its deficiency leads to the accumulation of mitochondria [22]. Recently, by searching for the AIM motif in mitochondrial proteins, 12 OMM proteins and 36 IMM proteins containing the AIM-like motif were predicted [12]. Of note, another recent screening study suggested that the Arabidopsis mitochondrial OMM Ca^2+^ channel, voltage-dependent anion channel 2 (VDAC2), is also predicted to harbor an AIM [54]. In Arabidopsis, defects in number and size of mitochondria were observed in most *vdac* mutants, but *vdac2* and *vdac4* mutant plants displayed a more severe growth retardation phenotype when compared with other *vdac* mutants, suggesting their possible distinct roles in plant growth [55,56]. In animals, it has been shown that VDAC2 is poly-ubiquitinated by Parkin and involved in mitochondrial quality control [24]. Plant PHBs, which are homologs of the mammalian IMM mitophagy receptor, may function as a candidate receptor in plant mitophagy. Loss of plant PHB3 leads to altered mitochondrial morphology and numbers [57]. A recent study has also reported that the deficiency in PHB3 reduces the levels of Salicylic acid (SA) but increases hypersensitive cell death in response to stress [58]. Nevertheless, the molecular link for plant PHBs in mitochondria or autophagy is still unclear. On the other hand, mutation of FtsH4 protease, which also locates to the IMM, causes mitochondria swelling and ROS accumulation [59]. Another study has further demonstrated that the autophagy-related defects, including leaf senescence caused by FtSH4, are dependent on the core ATG proteins [59]. It would be interesting to identify the mitochondrial substrates that are targeted by FtsH4 and to elucidate their functions in plant mitophagy. Taken together, future studies to explore the function of these OMM/IMM proteins, as well as their relationships with the ATG machinery during mitophagy should provide novel insights into the underlying mechanism of plant mitophagy.

### 2.3. Lipid-Dependent Mitophagy

Recently, a highly conserved IMM lipid cardiolipin (CL) was reported to recruit LC3 upon mitochondrial damage, suggesting that the lipid might serve as a targeting signal for mitophagy as well [40]. Under normal conditions, cardiolipin mediates IMM fusion together with OPA1 [60], but is exported to the OMM by the phospholipid scramblase-3 (PLS3) upon mitochondrial damage [40]. Another lipid named ceramide (CER), is synthesized into different forms by ceramide synthetase families (CerS) and released to the OMM to induce mitophagy as well [41]. It was observed that these CERs may promote the lipidation of LC3-PE to the OMM, and thus activate autophagy [41]. It was shown that the loss of cardiolipin synthetase in Arabidopsis leads to abnormal mitochondrial morphology, defective growth and reduced anti-stress response [5]. However, whether the lipid products are also exported to regulate mitophagy awaits further investigation in plants.

## 3. Interplay between Mitophagy and Mitochondrial Dynamics

### 3.1. Mitochondrial Fusion and Fission Machineries in Mitophagy

Mitochondrial fusion and fission is essential for maintaining the morphology and functions of healthy mitochondria. The following section will discuss different and conserved regulators in mammal and plant mitochondrial fusion/fission, as well as their interconnections with mitophagy (Table 1).

#### 3.1.1. Mitochondrial Fusion Machinery

In mammalian cells, OMM-located GTPases, MFN1/2 (mitofusin1/2), which will form homodimers or heterodimers to facilitate membrane tethering, play an essential role in mediating OMM fusion with adjacent mitochondria [71]. In addition, with other tethering factors, MFN1/2 forms a protein complex named ER-mitochondria encounter structure (ERMES) to mediate the fusion between ER membrane and mitochondria, also known as the ER-mitochondria contact (EMC). It has been observed that autophagosomal structures containing mitochondria were colocalized with ERMES, whereas disturbance of this complex affects the mitophagy level [72]. Thus, it has been suggested that the ER-mitochondria contact might provide membrane source to aid phagophore initiation and mitochondria sequestration. Another tethering complex, comprising mitochondria-localized VDAC1, inositol 1,4,5-triphosphate receptor 3 (IP3R) and ER-localized glucose-regulated protein 75 kDa (GRP75), mainly functions in the regulation of the intracellular Ca^2+^ homeostasis between ER and mitochondria [73]. VDAC1 has been previously identified as a Parkin substrate and ubiquitination of VDAC1 will further induce mitophagy, but it has also been suggested that other substrates rather than VDAC1 is responsible for the activation [24,74]. Interestingly, a recent study showed that Parkin promotes MFN2 phospho-ubiquitination, followed by MFN2 disassembly from the mitochondrial membrane to disrupt MFN2-mediated EMCs [75]. As a result, Parkin further enhanced ubiquitination VDAC1 and/or other mitochondrial substrates to facilitate the degradation of mitochondria, thus unveiling a novel molecular mechanism by interfering with EMCs via the Parkin-dependent ubiquitination of MFN2. However, FZO-like (FZL) protein, a homolog of MFN in plant, has been reported to function in plant chloroplast biogenesis, and no obvious defects in mitochondrial morphology was observed in *fzl* mutants [61].

On the other hand, the IMM fusion machinery also plays an essential role to maintain the architecture of mitochondria. Invagination of the IMM produces cristae, which requires optic atrophy 1 (OPA1) and the mitochondrial contact site (MICOS) complex [76]. OPA1 is a mitochondrial dynamin-like GTPase, which is processed by several proteases to produce the soluble short form (OPA1S) or the IMM-anchored form (OPA1L) [77]. Under severe stress conditions, only OPA1L participates in IMM fusion. Furthermore, when OPA1L is processed into OPA1S, IMM fusion is inhibited and mitochondrial fission is promoted [78]. Interestingly, OPA1 has also been shown to maintain the shape of mitochondrial cristae in a fusion-independent manner by antagonizing the Bcl2 associated protein X (BAX)-mediated apoptosis [79].

Recently, sorting assembly machinery 50 kDa subunit (SAM50), a β-barrel channel on the mitochondrial outer membrane, has been suggested to participate in mitophagy [80]. SAM50 forms a protein complex with MICOS to sustain the structural integrity of cristae [81]. Of note, the depletion of SAM50 induces fragmented mitochondria, which subsequently fused to form large abnormal spherical mitochondria [80]. By further interaction analysis, SAM50 was found to interact with PINK1 to regulate PINK1 stability. Moreover, it was also observed that the level of PINK1 was enhanced and Parkin-dependent mitophagy was accelerated in the SAM50 deficiency cells.

The majority of the mitochondria-related tethering machineries have not been identified in plants. By mitochondrial membrane proteome analysis, a list of novel plant proteins without any predicted mitochondria distribution were verified to distribute on the mitochondrial outer membrane, further suggesting that the mitochondrial tethering machinery might be diversified and specialized in plant species [82]. Recently, a plant-unique MICOS complex subunit, called DGD1 SUPPRESSOR1 (DGS1), has been found to regulate mitochondria and chloroplast morphology, as well as lipid homeostasis [83]. Particularly, the OMM-localized DGS1 serves as a bridge to link OMM-localized TOM40/20 and IMM-localized MIC60 [84]. However, orthologs of DGS1 are only conserved in yeast, but not in metazoan, implying a complexity in plant mitochondrial homeostasis during evolution. Nevertheless, a conserved protein in eukaryotic organisms, named FRIENDLY, which belongs to the CLUSTERED MITOCHONDRIA (CLUH) superfamily, has been reported to function in mitochondrial fusion in Arabidopsis [62]. It has been shown that mitochondria in *friendly* mutant cells formed large clusters. Interestingly, a recent study also uncovered a unique role of FRIENDLY in plant mitophagy [21]. In *friendly* mutants, abnormal mitophagosomes accumulated, and the level of mitophagy was significantly reduced upon mitochondrial uncoupler treatment, implying a defect in mitophagy due to the loss of FRIENDLY. Importantly, it was observed that Friendly overlapped with ATG8 upon mitophagy induction. This study has thus demonstrated an essential role of the mitochondrial fusion regulator in plant mitophagy for recycling damaged mitochondria during stress and development.

#### 3.1.2. Mitochondrial Fission Machinery

The mitochondrial fission machinery is conserved in most eukaryotes. Small GTPase including dynamin-related protein 1 (DRP1) in mammals and DRP3 in plants both have been shown to function as a driving force to segregate mitochondria [85]. When recruited to the OMM, DRP1 oligomerizes into a ring-like complex to strap the mitochondrial fission site, and ultimately segregates the mitochondrion [85]. However, DRP1 itself does not bind to the OMM directly, and is recruited by several mitochondrial receptors, respectively, including mitochondrial fission factor (MFF), mitochondrial elongation factor 1/2, also known as MiD51 and MiD49 (MIEF1/2) as well as mitochondrial fission 1 protein (FIS1), respectively [86]. Therefore, by interacting with a number of fission regulators, a high-order DRP1 complex is assembled on the mitochondrial surface to induce mitochondrial fission via its GTPase activity [87]. Mitochondrial fission, by isolating the damaged mitochondrial parts, has been generally regarded to make “bite-size” mitochondria prior to their engulfment by the autophagosome. However, a recent study suggested that the fission step might protect healthy mitochondria from degradation, likely by reducing the Parkin activity. It was also observed that loss of DRP1 triggered the recruitment of Parkin to mitochondria, and thus enhanced mitophagy activity [88]. Furthermore, comparing with *drp1* or *parkin* single mutation, the loss of both DRP1 and Parkin significantly compromised mitochondrial degradation, suggesting that both DRP1-dependent and DRP1-independent pathways are involved in mitophagy [89].

Indeed, it has been reported that mitochondrial division for mitophagy might be achieved by the core ATG machinery in coordination with autophagosome formation [90]. In particular, it was found that FIS1 interacts with syntaxin 17 (STX17), an EMC-related SNARE protein [91]. The FIS1-STX17 interaction relocates STX17 from ER onto mitochondria, subsequently recruiting other core ATG proteins, including ATG14. Loss of Fis1 induces the accumulation of STX17 on the mitochondria, which further enhances the formation of autophagosomes for mitophagy. It would be interesting to further investigate whether DRP1 and FIS1 participate in this process coordinately.

In Arabidopsis, there are two functional redundant orthologs of DRP1, named DRP3A and DRP3B, and both *drp3a* and *drp3b* mutants contain an abnormal elongated mitochondrial network [63]. The mitochondrial anchor for DRP1 is also conserved in Arabidopsis, including FIS1A (also known as BIGYIN) and FIS1B. Similar to the phenotype in the DRP3 mutant, deficiency of either FIS1A and FIS1B both caused arrested plant growth and abnormal clustered mitochondria [64,66]. Of note, elongated mitochondria 1 (ELM1) and peroxisomal and mitochondrial division factors (PMDs), were found to function as plant-unique fission factors in Arabidopsis. ELM1 is required for the relocation of DRP3 to the mitochondria, while PMD might regulate mitochondrial fission in a DRP3-independent manner, as a deficiency of PMD does not affect the DRP3 targeting the mitochondria [67,68]. Nevertheless, physical interaction for ELM1 with DRP3 or FIS1 has not been experimentally verified. It has been shown that the mutation of *elm1* reduced DRP3A/B recruitment to mitochondria when compared with the *bigyin* mutant in liverworts, suggesting ELM1 might function upstream of mitochondrial fission in plants [65].

In addition, the tethering machinery may coordinate with other trafficking regulators to reinforce membrane fission physically. It has been shown that mammalian DRP1 may interact with Bcl2-interacting factor 1 (BIF1), a BAR domain containing pro-apoptosis protein to mediate mitochondrial fission [92]. The BAR domain is able to induce membrane curvature by bending, thus providing a physical scaffold to facilitate the fission process [93]. Another recent publication has revealed that BIF1 interacts with both PHB2 and OPA1 in human cells to regulate mitochondrial dynamics and mitophagy [94]. Moreover, two Rho GTPases, mitochondrial Rho GTPase 1 and 2 (MIRO1 and MIRO2), were also suggested to participate in mitochondrial fission to mediate mitochondrial trafficking by bridging microtubules [95]. Loss of MIRO1 produced aggregated and thread-like mitochondria, suggesting impaired mitochondrial trafficking that hampered their fission. Recently, it was found that MIRO1 is degraded in Parkin-dependent mitophagy, which prevents the movement of damaged mitochondria to escape from clearance by autophagosomes [96]. Another recent study also reported that MIRO1 may interact with a low level of Parkin under normal conditions, but suppression of MIRO impaired Parkin translocation to mitochondria and suppresses mitophagy [97]. Counterparts of MIRO also exist in plants, with three isoforms in Arabidopsis. Although dysfunction of Arabidopsis MIRO1 caused embryo lethality, elongated mitochondria were detected in pollen tubes [69]. Interestingly, Arabidopsis MIRO2 knockout plants showed no obvious growth defects, but when there was overexpression of an active MIRO2 variant, larger and fewer mitochondria were observed in stable contact with the ER [70]. Contrary to the dominant function of MIRO in mitochondrial movement, MIRO might function in a plant-specific manner for both mitochondrial fusion and motility.

Taken together, multiple fusion/fission machineries participate in mitochondria dynamic and degradation (Table 1). Regarding the conservation and divergence of these fission/fusion machineries in plants, future investigation is required to further dissect their interaction relationships in plant mitochondria dynamic and mitophagy.

### 3.2. AMPK/SnRK1: A Master Energy Sensor in Balancing Mitophagy and Mitochondrial Dynamics

**Figure 2:**
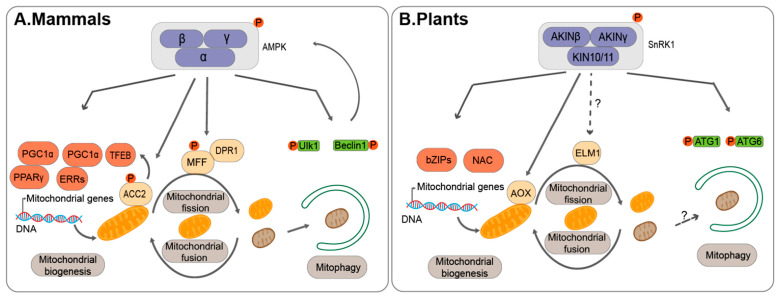
Regulation of mitochondrial homeostasis by AMPK/SnRK. (**A**) In mammals, upon energy stress, AMPK phosphorylates several transcription regulators essential for mitochondrial biogenesis gene expression, including peroxisome proliferator-activated receptor-γ (PPARγ) co-activator 1α (PGC1α), which activates mitochondrial biogenesis genes through interaction with PPARγ or oestrogen-related receptors (ERRs) [99,100,101]. Meanwhile, the transcription factor EB (TFEB), is also activated by AMPK to further induce PGC1α expression upon binding to their promoter region [102]. Mitochondrial outer membrane proteins, including Acetyl-CoA carboxylase 2 (ACC2) and mitochondrial fission factor (MFF), have also been identified as AMPK substrates [103,105]. In addition, AMPK also activates core ATG genes (e.g., ULK1, and Beclin1), which further phosphorylates downstream regulators in mitophagy [106,107,108,109,110,111,113,114,115,116,117,118]. (**B**) In plants, SnRK1 phosphorylates ATG1 and ATG6 [121,122,123]. In addition, SnRK1 also activates NAC transcription factor (NAC) and basic leucine zipper transcription factors (bZIPs), which are involved in regulating mitochondrial gene expression [124,125,126,127]. However, whether SnRK1 phosphorylates mitochondrial fusion/fission regulators (e.g., elongated mitochondria1, ELM1) is still unknown (dashed lines).

In addition to a tight coordination between mitochondria fission/fusion machineries and mitophagy regulators, upstream signal molecules also play important roles in coordinating mitochondrial dynamics and mitophagy activity. As one conserved key energy sensor, AMP-activated kinase (AMPK)/sucrose nonfermenting 1 (SNF1)/Snf1-related kinase1 (SnRK1), represents one of the upstream sensors in response to the cellular energy status, and subsequently phosphorylating various substrates to reprogram the cellular metabolism activities to balance mitochondrial biogenesis, mitochondrial fission/fusion and mitophagy [98] (Figure 2).

Firstly, it has been reported that several transcription factors essential for mitochondrial biogenesis are phosphorylated by AMPK in mammals. For example, AMPK phosphorylates peroxisome proliferator-activated receptor-γ (PPARγ) co-activator 1α (PGC1α), which activates mitochondrial biogenesis genes through interaction with PPARγ or oestrogen-related receptors (ERRs) [99,100,101]. On the other hand, AMPK also activates the transcription factor EB (TFEB), which further induces the expression of PGC1α upon binding to their promoter regions [102]. In addition, Acetyl-CoA carboxylase 2 (ACC2), a mitochondrial outer membrane protein involved in lipid metabolism, has also been found to be a substrate of AMPK. Of note, it has been suggested that ACC2 might enhance TFEB target gene expression upon phosphorylation by AMPK, suggesting positive feedback upon AMPK phosphorylation [103,104]. Moreover, it has been reported that AMPK might phosphorylate the mitochondrial outer-membrane receptor MFF, to control the number of mitochondria [105]. The activated MFF then recruits DRP1 to regulate mitochondrial fission process [105].

Particularly, AMPK may regulate mitophagy activity to control the degradation of mitochondria via direct phosphorylation of the core ATG proteins, including ULK1 Kinase. Upon mitochondrial oxidative stress, it has been shown that phosphorylation of AMPK and ULK1 were both increased [106,107]. Then, AMPK-dependent activation of ULK1 kinase facilitates its recruitment to the mitochondria, and further phosphorylates other downstream regulators, such as ATG9, which is suggested to transport the lipids for autophagosome formation, and small GTPase Rab9 for the transport of damaged mitochondria into the lysosome [108,109]. In addition, it was also shown that ULK1 phosphorylates mitophagy receptors to promote mitophagy [110,111]. Another key ATG protein that has been implicated in mitophagy is Beclin1 (ATG6 homolog), which forms different class III PI3K complexes for either intracellular vesicle trafficking or autophagy [112]. Interestingly, it was found that AMPK facilitates the phosphorylation of Beclin1 to form the autophagy PI3K complex to induce autophagy [113,114]. Beclin1 directly interacts with the protein kinase PTEN-induced kinase 1 (PINK1) to aid in the translocation of the E3 ligase Parkin to mitochondrial membrane to induce mitophagy [115,116]. However, anti-apoptotic protein B-cell lymphoma 2 (Bcl-2) binds to Beclin1 to negatively regulate the pro-survival role of autophagy [117]. In addition, a recent study showed that energy stress enhanced Beclin1 interaction with a Toll-like receptor 9, which further activated AMPK activity but inhibited Beclin1-Bcl-2 interaction [118], thus providing another positive feedback loop to enhance autophagy for cell survival.

In plants, the Sucrose Nonfermenting-Related Kinase 1 (SnRK1) family serves as the counterpart of AMPK as an energy sensor and actively participates in plant stress response [119]. Of note, it has recently been shown that SnRK1 subunit KINβγ regulates the biogenesis of mitochondria to monitor ROS levels in pollen [120]. A mutation of *kinβγ* leads to abnormal mitochondrial biogenesis, as well as reduced ROS production. However, the plant SnRK1 regulation mechanism might be different from that in mammals, as downregulation of KIN10/11 and KINβγ reduced the number of mitochondria, while more mitochondria are observed in AMPK defective mouse liver cells. Nevertheless, ATG1 proteins have also been shown to be phosphorylated by the catalytic α-subunit of SnRK1, KIN10 in Arabidopsis [121,122]. Recently, it was also reported that KIN10 can phosphorylate ATG6 to promote autophagy [123]. In addition, an NAC transcription factor, named suppressor of gamma response 1 (SOG1), was identified to interact with SnRK1 in response to low energy [124]. Moreover, both *sog1* and *kin10*/*kin11* mutant can partially restore a mitochondrial mutant *sd3*, which contains low abundant ATP due to a defect in a mitochondrial inner membrane protein. Another example is the regulation of the alternative oxidase (AOX), a key enzyme involved in the electron transport chain in mitochondria. KIN10 may coordinate with cyclin-dependent kinase E1 (CDKE1) to induce AOX1a, likely via NAC17 transcription factor [125,126]. On the other hand, it was reported SnRK1 might target one subfamily of basic leucine zipper transcription factors (bZIPs), S_1_-bZIPs, which also participates in regulating mitochondrial respiratory genes [127]. However, whether other mitochondrial fission/fusion regulators are also targeted by SnRK1, such as those in mammals, remains unclear (Figure 2). Nevertheless, it seems that multiple layers of regulation in mitochondrial dynamics and mitophagy by AMPK/SnRK1 are conserved in both mammals and plants in mitochondrial quality control.

## 4. Interplay between Mitophagy and PCD: A New Role of the BAG Protein Family

Although mitophagy and mitochondrial fission may aid in the clearance of harmful/unwanted mitochondrial material, exactly how these quality-control pathways sense different levels of mitochondrial damage to ensure proper execution remains poorly understood. Upon stress induction and mitochondrial damage, the MOMP is increased, accompanied by the loss of ΔφM, which will further activate PCD [7,128]. This raises another important fundamental question as to how mitophagy and PCD are balanced for cell fate decisions, particularly at the molecular level. In animals, the versatile Bcl-2 protein family functions as a key regulator for PCD [129,130]. There are both pro-survival and pro-death Bcl-2 members reported in animal PCD. In response to stress or developmental signals, pro-death Bcl2-associated protein x (BAX) forms a Cytochrome c channel to facilitate the release of the latter, while pro-survival proteins Bcl-2 and Bcl-XL can either suppress the downstream Caspase or the release of Cytochrome c, respectively, for downstream execution of PCD [130,131]. However, no counterparts of Bcl-2 protein have been identified in the plant genome.

**Figure 3:**
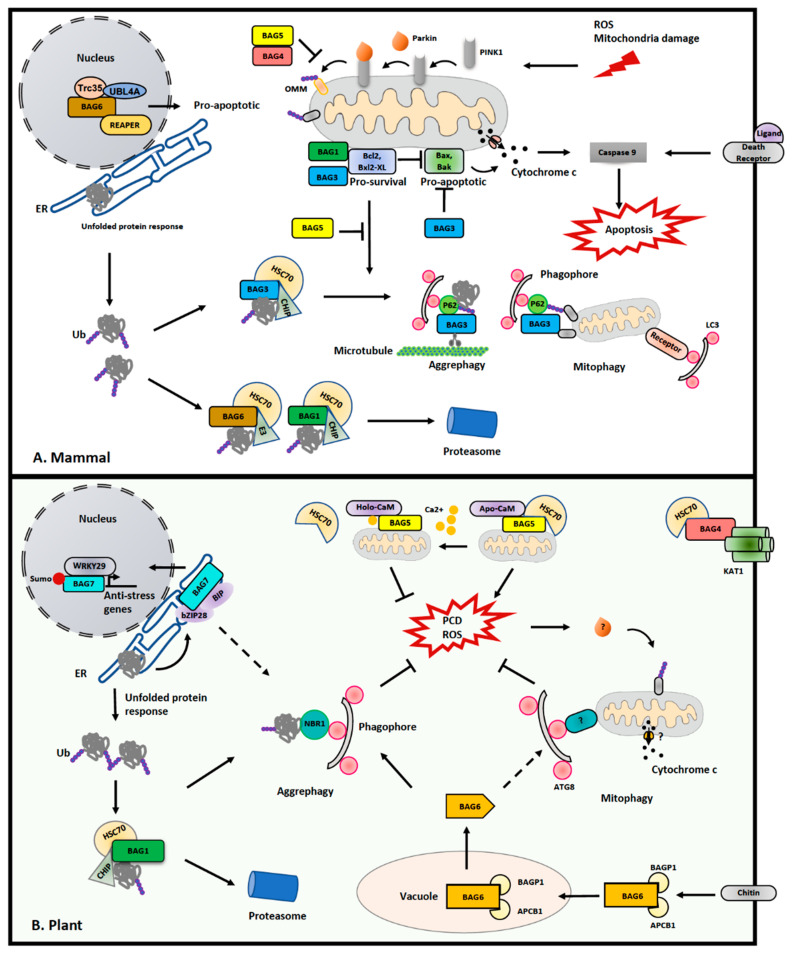
Functions of BAG families in mitochondrial homeostasis and PCD in mammals and plants. (**A**) Balance between anti-apoptotic and anti-survival Bcl-2 proteins determines the levels of the pro-death and pro-survival pathways. In addition, 6 isoforms of BAG proteins are coded in mammals, among which BAG1 mainly participates in unfolded protein response [133,134]; BAG3 induces autophagy by competing with BAG1 and binding with p62 as well microtubule regulator, or by directly binding with OMM protein to promote autophagy [135,136,137,138]; BAG4 and BAG5 both inhibit mitophagy by suppressing Parkin activity and pro-survival pathway [139,140]; BAG6 forms a ternary complex in the nucleus to promote pro-apoptotic activity via REAPER, and also participates in proteasome-mediated unfolded protein response [141]. (**B**) In plants, although no homologues of Bcl-2 proteins have been identified, seven isoforms of BAG proteins are found, which have diverse distributions. BAG1-3 has been implicated in binding to HSC70, mediating the unfolded protein response [132,142,143,144]; BAG4 has been shown to interact with the potassium channel KAT1 to participate in stomatal movement [145]; BAG5 is a mitochondrial-localized protein, whose activity is switched by Ca^2+^ levels via regulating its binding affinity towards CaM and Hsc70 [146]; BAG6 requires a proteolytic process in the vacuole for its activation, which participates in pathogen response and autophagy as well [147,148]; BAG7 is an ER-localized protein, which forms a complex with Hsc70 ortholog BIP protein and bZIP28 upon ER stress [149]. Additionally, after cleavage, BAG7 will undergo SUMOylating and tranlocation to interact with the WRKY29 in the nucleus to regulate the expression of several anti-stress genes [150].

Although no Bcl-2 counterparts have been identified in plants, the Bcl-2-associated athanogene (BAG) family proteins, which were initially discovered as Bcl-2-interacting partners to participate in PCD regulation, are remarkedly conserved in plant genomes [132]. Of note, recent studies in animals and plants implied a crucial role of the BAG proteins in mitochondrial homeostasis, PCD and stress response (Figure 3). Importantly, several BAG proteins have been implicated to negatively or positively regulate Parkin-mediated mitophagy [133]. As cochaperones binding to heat shock proteins heat shock protein 70 (Hsp70), BAGs probably serve as an “off-on” switch mechanism between PCD and mitophagy by bridging various molecular chaperones and target proteins, thus balancing the level of PCD and mitophagy.

There are six BAG proteins identified in animals, and all of them contain at least one BAG domain for binding to the ATPase domain of Hsc70 [134,151]. As the first identified Bcl-2 associated BAG protein, BAG1 has been extensively studied. BAG1 contains an N terminus ubiquitin-like domain (UBL) to form a complex with Hsc70, and an E3 ligase named C terminus of Hsc70-interacting protein (CHIP) for proteasome-mediated degradation [134,151]. Differently, BAG3 comprises a tryptophan–tryptophan (WW) domain and PxxP motif, both of which bind to proline-rich proteins. In particular, BAG3 harbors two additional IPV (Ile-Pro-Val) motifs for recruiting specific HSP70, and links the BAG3-containing protein aggregates via the autophagic adaptor p62 [136]. Interestingly, it has been suggested that a shift in binding with Hsc70 from BAG1 to BAG3 may serve as a switch-off mechanism to balance proteasome-dependent proteolysis and selective autophagy [137]. Another piece of evidence showed that BAG3 interacts with an actin-binding protein Synaptopodin, named SYNPO, to facilitate the clearance of microtubule-associated protein tau (MAPT), further supporting the specific role of BAG3 in selective autophagy [138]. Recently, another study also demonstrated that BAG3 may promote mitophagy by recruiting Parkin to the depolarized mitochondria [135]. Conversely, BAG4 and BAG5 have been implicated in disturbing the translocation of PARKIN to the mitochondria, thus suppressing mitophagy [139,140]. Of note, BAG5 directly binds to Parkin to suppress its E3 ligase activity for mitophagy, but promotes Parkin-mediated proteolysis degradation for PCD upon depolarization of mitochondria, indicating a role of BAG5 in switching the pro-survival and pro-death activity of Parkin [139]. Differently, the nucleus-localized BAG6 might trigger a cell death pathway via binding to the apoptosis inducer REAPER, and it also regulates the unfolded protein response for protein quality control in the cytosol [141].

**Figure 4:**
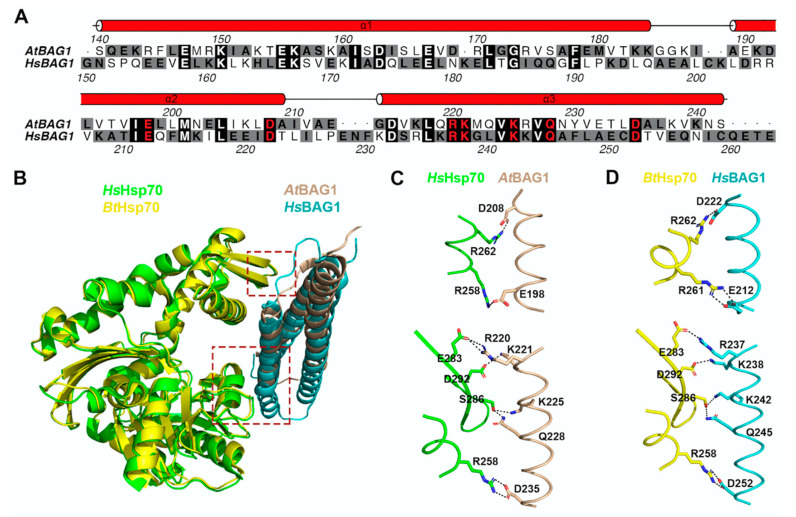
Conserved interactions with Hsp70 for Arabidopsis BAG1 or human BAG1. (**A**) Sequence alignment of BAG domain from *Arabidopsis thaliana* and *Homo sapiens* BAG1 protein. Secondary structure and residue numbers were numbered according to Arabidopsis BAG1. Conserved residues forming the interaction surface with the Hsc70 are highlighted in red. (**B**) Structure comparison of complex of Hsp70 with BAG domain from Arabidopsis (PDB code 4HWI) and humans (PDB code 1HX1). (**C**) Enlarged schematic diagram of the interactions between BAG domain of Arabidopsis BAG1 and Human Hsp70 from B. (**D**) Enlarged schematic diagram of the interactions between BAG domain of human BAG1 and *Bos taurus* Hsp70 from B.

In *A. thaliana*, seven BAG proteins have been predicted based on their consensus BAG domain, and are distinguished into two groups [132]. Arabidopsis BAG1-4 share similar structures as human BAG1, which comprise an N-terminal UBL domain and a C-terminal BAG domain [129], likely playing a redundant role in Arabidopsis. Differently, BAG5-7 contain a plant-unique CaM (calmodium) binding motif (IQ) as well as the BAG domain, but lack the UBL domain [129]. It is implicated that all Arabidopsis BAGs are associated with Hsp70, except BAG6 [132,142]. Structural analysis has also revealed that Arabidopsis BAG1 and human BAG1 both use conserved residues within their BAG domain for interacting with Hsp70 (Figure 4) [143]. Moreover, recent progress has unveiled important roles of the BAG protein family in the plant stress response. For example, BAG1 has been reported to participate in the degradation of unimported plastid proteins, together with Hsp70 and CHIP, and overexpression of BAG1 impairs plant development and response to high salt conditions [142]. In another study, it was shown that BAG2 deficiency promotes plant growth [144]. However, both *bag4* and *bag6* mutant plants displayed an early senescence and higher susceptibility to salt stress [132]. All these data support that the level of BAG proteins is critical in plant development and stress response. Recently, through yeast two hybrid screening, it was also reported that BAG4 interacts with KAT1 potassium channel in mediating stomatal opening [145]. Regarding the essential role of stomata opening in drought tolerance and pathogen susceptibility, the BAG4–KAT1 interaction might function as a posttranslational regulation mechanism to participate in plant stress response.

Among all the Arabidopsis BAG proteins, only BAG5 has been reported to localize on the mitochondria, and structural analysis suggests a role of BAG5 in the spatiotemporal regulation of plant senescence [146]. Under normal conditions, BAG5 binds to CaM and Hsp70 independently [146]. However, when the mitochondrion is damaged and the matrix Ca^2+^ is released, the high Ca^2+^ induces a change in the binding mode for CaM with BAG5, which inhibits BAG5 binding to Hsp70 to enhance ROS level, and finally leads to senescence. It has also been shown that no significant developmental defect was observed in the *bag5* mutant. Conversely, overexpression of BAG5 or its IQ domain accelerated leaf senescence, and senescence-associated genes (SAGs) were also upregulated. The direct evidence that links BAG protein in autophagy comes from recent studies of BAG6. It was reported that BAG6 triggers autophagy upon fungal infection [147]. Further analysis showed that the invading fungi insert their chitin hypha into the infected plant cell, which subsequently leads to the vacuolar cleavage of BAG6 on a specific caspase-1 site via binding to a C2 GRAM domain protein (BAGP1) and an aspartyl protease (APCB1) [148]. The truncated BAG6 then activated autophagic response to the pathogen, although the substrate for degradation is unknown [148]. Significantly, mutations on the BAG6 cleavage site suppressed autophagy in plants and inhibited disease resistance, implying that proteolytic activity is also required for BAG6 to execute its pro-survival function [148].

Compared with other BAGs, BAG7 has a unique distribution on the ER, and has been shown to interact with BIP2 in the bZIP28-mediated unfolded protein response (UPR) upon heat and cold stress [149]. An essential role of BAG7 in ER-induced cell death is supported by the observation that cell death is accelerated in *bag7* mutants [149]. It was proposed that BAG7–BIP2 interaction might facilitate the retention of bZIP28 on the ER, whereby BAG7 undergoes proteolytic cleavage and is translocated into the nucleus under stress conditions [149]. After further SUMOylation modification, BAG7 interacts with the transcription factor WRKY DNA binding protein 29 (WRKY29) to mediate chaperone expression, while bZIP028 interacts with bZIP60 to activate UPR gene expression [150]. Therefore, BAG7 also functions as a “switch-on” signal for cell death. Surprisingly, an AIM-like motif is predicted in BAG7 [54]. Thus, it will be interesting to determine, in the future, whether BAG7 is a target of ATG8 to balance between cell death and cell survival. Furthermore, another study also unveiled an unexpected anti-survival role of BAX inhibitor 1 (BI1) in bZIP28-mediated pro-survival signaling in ER stress recovery. It has been shown that sensitivity to ER stress is partially recovered in the *bzip28 bi1-2* double mutant when compared with *bzip28* in roots, suggesting that BI1 may antagonize the effect of bZIP28 during ER stress recovery [152]. In addition, Arabidopsis BI1 also binds to ATG6, and the suppression of BI1 expression impaired the formation of autophagosomes upon viral infection, while its overexpression caused autophagy-dependent cell death [153]. Taken together, although plant genomes lack BCL-2 members, tremendous progress has been made to unveil a diversification of BAG proteins for the switching of cell fate in a plant-specific manner. Future efforts are required to further identify the BAG network to reveal their specific roles in balancing plant organelle quality control, particularly the mitochondria, and PCD.

## 5. Conclusions

Both mitochondrial biogenesis and mitochondrial degradation are essential for maintaining mitochondrial homeostasis. Mitophagy, as a key degradation process for the clearance of unwanted mitochondria, has been intensively studied in animal models for its close involvement with several diseases. Compared with other systems, it is obvious that there are lots of questions unsolved in plant mitophagy: What are the receptors linking the autophagosome and the mitochondrion under different conditions in plants? How do the mitochondrial fission/fusion machineries coordinate with the ATG machinery to facilitate mitophagy in plants? How is plant PCD executed to antagonize mitophagy under stress conditions? Future exploration into the molecular network for linking mitochondrial fission/fusion machinery, mitophagy and PCD should provide us with a better understanding of how mitochondrial homeostasis is maintained for plant development and stress response.

## Figures and Tables

**Table 1:** Key mitochondrial fusion and fission factors *.

	*Hs*	*Sc*	*At*	Function in Mitochondrial Dynamic	Phenotype in Plants	References in Plants
**Fusion**	MFN1/2	FZO1	FZL	Mitochondria-organelle contacts	Abnormal chloroplast and thylakoid morphology	[61]
	OPA1	-	-	Inner mitochondrial membrane fusion; Cristae shape maintenance	-	-
	IP3R3	-	-	ER mitochondria contacts	-	-
	VDAC1	POR1	VDAC1-6		Swollen and fewer mitochondria	[55,56]
	GRP75	-	-		-	-
	SAM50	SAM50	-	Cristae shape maintenance	-	-
	CLUH	CLU1	FRIENDLY	Mitochondrial fusion	Clustered but not fused mitochondria; defective in mitophagy	[21,62]
**Fission**	Drp1	DRP1	DRP3A; DRP3B	Mitochondrial segregation	Elongated, networked mitochondria	[63,64,65]
	FIS1	FIS1	FIS1A (BIGYIN); FIS1B	Recruitment of dynamins to the outer mitochondrial membrane	Enlarged and fewer mitochondria	[64,66]
	MFF	-	-		-	-
	MIEF1/2 (MiD49/51)	-	-		-	-
	-	-	ELM1		Elongated and fewer mitochondria	[65,67]
	-	-	PMD1; PMD2	DRP3-independent mitochondrial segregation	Elongated mitochondria	[68]
	BIF1	-	-	Membrane curvature	-	-
	MIRO1/2	-	MIRO1/2/3	Mitochondrial movement; ER-mitochondria contacts	Elongated mitochondria in *miro1* mutant; Few ER-mitochondria contacts in *miro2* mutant.	[69,70]

* Not all regulators are listed. Abbreviations: Hs, *Homo sapiens*; Sc, *Saccharomyces cerevisiae*; At, *Arabidopsis thaliana*.

## Data Availability

Not applicable.
